# Potential diagnostic markers and therapeutic targets for periodontitis with comorbid infective endocarditis based on bioinformatics and experimental analyses

**DOI:** 10.3389/fcell.2025.1725544

**Published:** 2025-11-20

**Authors:** Feng Mei, Wenjie Zhang, Xinlin Wang, Yutong Liu, Xiangyu Zhou, Ting Zhou, Wei Zhang

**Affiliations:** 1 College & Hospital of Stomatology, Anhui Medical University, Key Laboratory of Oral Diseases Research of Anhui Province, Hefei, China; 2 Hubei Minzu University, Enshi, Hubei, China; 3 Department of Cardiology, Renmin Hospital of Wuhan University, Wuhan, Hubei, China; 4 Department of Obstetrics and Gynaecology, The First Affiliated Hospital of Anhui Medical University, Hefei, Anhui, China

**Keywords:** infective endocarditis, periodontitis, hub gene, bioinformatics analysis, immune cell subtype relative proportion analysis

## Abstract

**Background:**

Periodontitis (PD) and infective endocarditis (IE) significantly impair quality of life and contribute to considerable socioeconomic burdens. Accumulating evidence supports a bidirectional interaction between these diseases that aggravates clinical outcomes. This study aims to identify diagnostic biomarkers and investigate therapeutic targets underlying PD-IE comorbidity.

**Methods:**

We analyzed disease-specific differentially expressed genes (DEGs) for PD and IE from datasets in the Gene Expression Omnibus (GEO). Functional enrichment analysis was then performed to investigate the biological roles of the DEGs in PD and IE datasets. Protein-protein interaction (PPI) networks were constructed using STRING database and hub genes were identified utilizing cytoHubba plugin in Cytoscape software. We selected diagnostic markers using a dual-algorithm approach that combined differential expression analysis and receiver operating characteristic (ROC) curve analysis, and subsequently evaluated their immune associations and therapeutic relevance. Our key findings were further validated using *in vivo* PD models.

**Results:**

A total of 22 DEGs were identified as common to both PD and IE. PPI analysis uncovered five hub genes. ROC curve analysis supported the diagnostic utility of these five markers. Following expression analysis, we identified 4 hub genes as potential diagnostic markers: ITGAM, FCGR3B, FCGR3A and ITGB2. Significant correlations were observed between the expression of these genes and immune cell infiltration in both diseases. *In vivo* experiments confirmed the upregulation of the potential diagnostic markers in PD tissues compared to normal controls.

**Conclusion:**

ITGAM, FCGR3B, FCGR3A, and ITGB2 can serve as mechanistically informative diagnostic biomarkers and promising therapeutic targets for PD-IE comorbidity. The shared pathophysiology of the two diseases involves immune response mechanisms. Our findings open up new avenues for the concurrent therapeutic targeting of both diseases.

## 1 Introduction

Periodontitis (PD), an inflammatory disease of tooth-supporting tissues induced by oral bacteria, affects approximately 19% of the global population over 15 years (>1 billion cases), ranking among the most prevalent non-communicable diseases (Schulze-Späte et al., 2024). Its development is modulated by host responses to dysbiotic oral microbiota. PD independently associates with all-cause mortality and post-cardiovascular event mortality (Romandini et al., 2021). Clinically, PD exhibits a strong association with infective endocarditis (IE)-a life-threatening systemic infection of the cardiac endothelium, frequently involving valves, that carries substantial morbidity and mortality (Liu et al., 2025). Contemporary IE epidemiology increasingly affects patients with significant comorbidities, structural heart disease, cardiovascular devices, or healthcare-associated infections (Lau et al., 2025). Notably, *staphylococcus* aureus-associated IE with comorbid PD has risen, a concerning trend given this pathogen’s association with poorer outcomes. Shared risk factors (type II diabetes, chronic stress, smoking, obesity) provide incomplete explanation for the multifaceted PD-IE interactions (Schulze-Späte et al., 2024). The precise biological crosstalk between these diseases remains incompletely characterized.

Previous Mendelian randomization analyses have demonstrated the absence of causal links between PD and atrial fibrillation, stroke, coronary heart disease, or heart failure (Czesnikiewicz-Guzik et al., 2019; Zhou et al., 2021; Carrizales-Sepúlveda et al., 2018), but indicate a potential causal association with both hypertension and IE (Carinci et al., 2023). A cross-sectional study conducted by Ninomiya revealed that PD patients with IE had more advanced bone resorption, fewer remaining teeth when compared with those without IE, which confirmed a significant association between PD and the occurrence of IE (Ninomiya et al., 2020). It has been reported that PD is characterized by inflamed and ulcerated gingival crevicular epithelium, which facilitates bacterial translocation from the oral cavity into the bloodstream. Besides, generalized gingival bleeding following tooth brushing has been associated with an approximately 8-fold increase in bacteremia risk (HO, 2006). This aligns with further evidence indicating that the incidence and extent of bacteremia induced by mastication, oral hygiene activities, and invasive dental procedures are correlated with PD severity (Lockhart et al., 2009). The earlier study showed that most cases of IE were initiated by hematogenous dissemination of microorganisms to cardiac tissues (Naderi and Merchant, 2020), and PD-induced inflammatory damage promoted vascular access, thereby enabling microbial transit to the heart and contributing to cardiovascular pathology (Drangsholt, 2024). While the link between PD and bacteremia, and the implications on IE, dates as far back as the early 1900s (Cahill et al., 2017), and a possible association between PD and IE has been more recently recognized (Dhotre et al., 2018), the exact mechanism between PD and IE has not been clearly determined.

In this research, gene microarray technology provided a powerful approach to identify potential therapeutic targets common to both PD and IE. Through integrated bioinformatics analysis, we conducted functional enrichment of differentially expressed genes (DEGs) obtained from PD- and IE-related datasets in the NCBI Gene Expression Omnibus (GEO). This enabled the identification of key molecular targets and pathways implicated in their shared pathogenesis. Protein-protein interaction (PPI) networks was further employed to identify hub genes which further selected by expression analysis. Additionally, receiver operating characteristic (ROC) curves were generated to evaluate the diagnostic value of these hub genes in PD and IE. We also evaluated immune cell infiltration patterns and explored the correlations between hub genes and immune cells across both diseases. Finally, we validated the expression and clinical relevance of the hub genes using quantitative real-time PCR (qRT-PCR) and Western blot (WB) analyses.

Given the accumulating evidence supporting a close association between PD and IE, we employed advanced bioinformatic analyses to identify diagnostic biomarkers for both diseases. This study is a novel exploration of the shared pathological mechanisms in PD and IE comorbidity and can offer new insights into the genetic underpinnings and potential therapeutic strategies for these conditions.

## 2 Materials and methods

### 2.1 Data acquisition and processing

**FIGURE 1:**
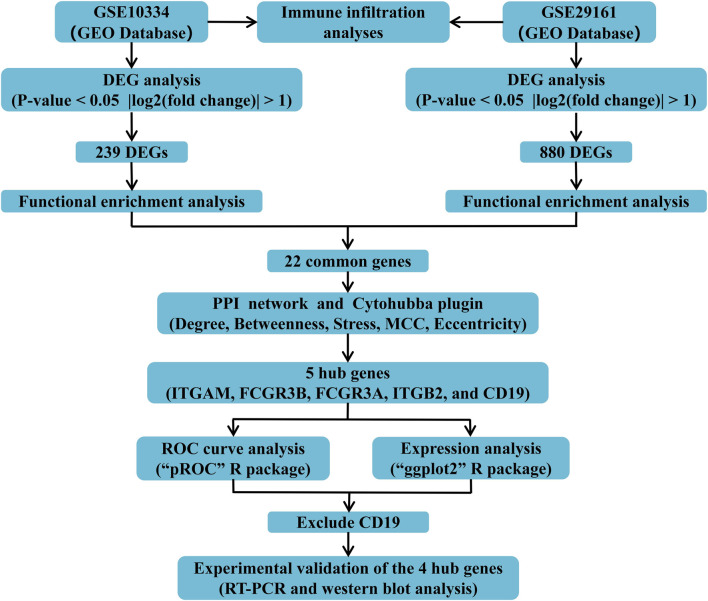
The flowchart presenting the overall study design.

The datasets utilized in this analysis were sourced from the GEO public data repository (https://www.ncbi.nlm.nih.gov/geo/). We applied the following inclusion criteria to screen the PD dataset: (1) human-derived origin, (2) gene expression profiled by microarray, (3) inclusion of both control and disease cohorts. We applied the following inclusion criteria to screen the IE dataset: (1) human-derived origin, (2) inclusion of cardiac valve tissues, (3) gene expression profiled by microarray, (4) inclusion of both control and disease cohorts. This study incorporated the following datasets: periodontitis-associated data (GSE10334): This dataset compared the transcriptomic profiles of diseased tissues from 64 patients with chronic PD to those from 63 healthy individuals. Gene expression was profiled using the GPL570 platform ([HG-U133_Plus_2] Affymetrix Human Genome U133 Plus 2.0 Array). Endocarditis-associated data (GSE29161): This dataset investigated transcriptomic differences between valvular tissues from 10 patients with IE and 5 healthy controls, profiled on the GPL6480 platform (Agilent-014850 Whole Human Genome Microarray 4 × 44K G4112F (Probe Name version)). Batch correction of the datasets was performed utilizing the ComBat method implemented in the sva package (v3.36.0) for R software (v4.0.2) to mitigate biases arising from combining samples processed in different batches. The flowchart was provided to illustrate the overall study design (Figure 1).

### 2.2 Principal component analysis (PCA)

PCA was conducted using the prcomp () function from the base R package. The results were visualized by generating a PCA plot with the ggplot2 package (v3.3.3) to illustrate the distributional differences between the Reference (Ref) and Test groups.

### 2.3 Differential gene expression analysis

Following PCA, differential expression analysis was performed on the PD and IE datasets using the limma package (v3.44.3). DEGs were defined as those with a |log_2_ (fold change)| >1 and a p-value <0.05. Subsequently, volcano plots and heatmaps were generated using the ggplot2 package (v3.3.2) and the pheatmap package (v1.0.12), respectively, to visualize the expression patterns of the DEGs.

### 2.4 Functional enrichment analysis

Gene Ontology (GO) enrichment analyses were conducted separately on the DEGs in PD and IE datasets using the Metascape database (v3.5). Enrichment analysis was conducted with the following thresholds: a p-value <0.01, minimum overlap count = 3, and minimum enrichment factor = 1.5. The results were visualized using bar charts and the network diagrams of enriched GO terms were further generated to reveal the relationships between the individual terms.

### 2.5 PPI network construction and analysis

After identifying DEGs in PD and IE, a Venn diagram was generated using the VennDiagram package (v1.7.3) to visualize and identify the intersection of genes commonly associated with both conditions. Using STRINGdb (v2.6.1) and igraph (v1.4.0), a PPI network was constructed and visualized with a minimum interaction score threshold of 0.400. Interaction data were retrieved from the STRING online database (v10.5) and imported into Cytoscape software (v3.10.2) for topological analysis. The cytoHubba plugin (v0.1) was utilized to determine the top 10 hub genes within key clusters based on centrality metrics including Degree, Betweenness, Stress, Maximal Clique Centrality (MCC) and Eccentricity, enabling the screening of core regulatory genes. These specific metrics were chosen as they collectively provide a multi-faceted assessment of a gene’s importance within the network by measuring direct connectivity (Degree), control over information flow (Betweenness), communication burden (Stress), robustness in dense modules (MCC), and propagation efficiency (Eccentricity), thereby ensuring a comprehensive identification of core regulatory genes.

### 2.6 Boxplot visualization of gene expression

To visualize the differential expression of hub genes, we generated boxplots comparing healthy controls and disease cohorts. These plots were created using the ggplot2 package (v3.4.0).

### 2.7 ROC curve analysis

In this study, ROC curve analysis was employed to evaluate the diagnostic performance of hub genes. Initially, hub genes were selected and used to construct binary classification models based on their expression levels. Subsequently, we utilized the roc () function from the pROC package (v1.18.0) to calculate sensitivity (true positive rate) and specificity (1 - false positive rate) for each gene, followed by ROC curve generation. Finally, the overall predictive power was quantified by computing the Area Under the Curve (AUC) metric. AUC values approaching 1 indicate superior discriminatory power of the model. Visualization of ROC curves was performed using the ggplot2 package (v3.3.2).

### 2.8 Analysis of immune cell subtype relative proportions

The relative proportions of immune cell subtypes were analyzed using CIBERSORT (v1.03), a deconvolution algorithm that infers immune cell composition from gene expression data. The analysis was performed using the standard CIBERSORT workflow with the LM22 signature matrix. The pre-processed gene expression matrix was subjected to the CIBERSORT function within the CIBERSORT package to calculate subtype abundances across all samples. Using ggplot2 (v3.4.0), we generated stacked bar plots to illustrate the compositional proportions of immune cell subsets in each sample, and box plots to compare their relative abundance across different groups. To investigate the correlations among immune cells, we also generated a correlation heatmap of immune cells. Spearman’s rank correlation coefficients between hub genes and infiltrating immune cell proportions were computed for both the PD and IE datasets using the cor. test function in R, with data arrangement facilitated by the dplyr (v1.0.7) package. Subsequently, the results were visualized using lollipop plots generated with ggplot2 (v3.4.0) to display the strength of correlations between immune cell types and gene expression.

### 2.9 Construction of the PD model

C57BL/6 wild-type mice were acquired from the Experimental Animal Center after receiving approval from the Animal Care and Use Committee of Anhui Medical University. Animals were maintained under specific pathogen-free conditions with a 12-h light/dark cycle. PD was induced using a method wherein 5–0 silk ligatures were secured around bilateral maxillary second molars for 6 weeks. Control mice received no ligatures. At experimental endpoints, mice were sacrificed and maxilla tissues were harvested for analysis.

### 2.10 Hematoxylin–eosin (HE) staining

Maxillary specimens were fixed in 4% neutral buffered formalin for 24 h, followed by decalcification in 15% EDTA (pH 7.4) for 2 weeks with solution replacement every 48 h. Following embedding in paraffin wax, the tissues were cut into 5 µm-thick sections. Serial sections (5 µm thickness) were stained with HE staining to assess general tissue morphology. The images were captured through an inverted microscope.

### 2.11 Western blot analysis

RIPA buffer supplemented with protease and phosphatase inhibitors was used to extract proteins from the maxillary tissues. A BCA assay kit (P0012, Beyotime Biotechnology) was employed to measure protein concentrations. Following separation by SDS-PAGE, proteins were transferred to PVDF membranes (Millipore). After blocking, membranes were incubated with the following primary antibodies: anti-ITGAM (Sc20050; Santa Cruz), anti-ITGB2 (Sc8420; Santa Cruz), anti-FCGR3B (A7894, ABclonal), anti-FCGR3A (33531-1-AP, Proteintech), anti-β-actin (66009-1-Ig, Proteintech). HRP-conjugated secondary antibodies were then applied. Protein bands were visualized using Western Bright ECL substrate, with β-actin serving as the loading control.

### 2.12 Real-time polymerase chain reaction (RT-PCR) analysis

Maxillary tissues were pulverized in liquid nitrogen, and total RNA was extracted with TRIzol Reagent (Invitrogen, United States). Target gene mRNA levels were quantified via quantitative PCR with SYBR^™^ Premix Ex Taq^™^ II. Relative expression was calculated using the 2^−ΔΔCT^ method, with GAPDH as the housekeeping control. Primer sequences were as follows:

*Itgam*: Forward: 5′-TCTTCTGGTCACAGCCCTAG-3’; Reverse: 5′-CTGGACCACACTCTGTCCAAA-3’.

*Itgb2*: Forward: 5′-TTGCTGCATGTCCGGAGGAA-3’; Reverse: 5′-GTCAGGATGGCACCCAGTTT-3’.

*Fcgr3a*: Forward:5′-GCATTCAAGCTGGTCTCCAA-3’; Reverse: 5′-GGTCACGCTGTCTTCCTCAA-3’.

*Gapdh*: Forward:5′-GGCTCATGACCACAGTCCAT-3’; Reverse: 5′-CCATCACGCCACAGCTTTC.

### 2.13 Statistical analysis

Bioinformatics analyses were performed using R (v4.4.1). RT-PCR and Western blot data represent mean ± SEM and were analyzed in GraphPad Prism 8. Normality was verified by Shapiro-Wilk test, with between-group differences assessed using unpaired Student’s t-tests. *P < 0.05; **P < 0.01; ***P < 0.001; ****P < 0.0001.

## 3 Result

### 3.1 Data acquisition and processing

**FIGURE 2:**
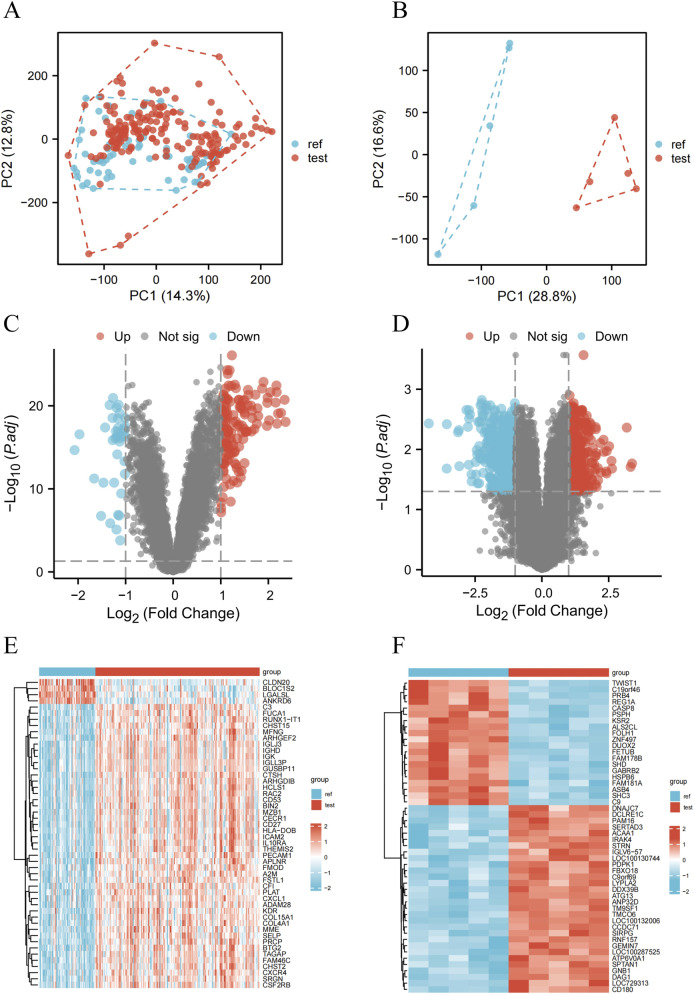
Identification of differentially expressed genes (DEGs). **(A,B)** PCA plots showing significant separation between groups along the first two principal components (PC1 and PC2). **(C,D)** Volcano plots depicting DEGs for datasets PD (GSE10334) and IE (GSE29161), respectively. Significantly upregulated genes were shown in red, significantly downregulated genes in blue, and non-significant genes in grey. **(E,F)** Heatmaps illustrating the expression patterns of representative significantly upregulated and downregulated DEGs identified in PD (GSE10334) and IE (GSE29161) datasets, respectively.

Human gene expression datasets GSE10334 (PD) and GSE29161 (IE) were retrieved from the NCBI GEO database. To assess data quality, PCA analysis was performed. The results showed clear separation between samples in both PD and IE datasets, indicating high reproducibility and dataset quality (Figures 2A,B).

### 3.2 Identification of DEGs in PD and IE datasets

To identify key genes in the PD and IE datasets, we applied a screening threshold of |log_2_ (fold change)| >1 and *p* < 0.05. Specifically, the PD dataset exhibited 239 DEGs, the IE dataset exhibited 880 DEGs. Volcano plots (Figures 2C,D) and heatmaps (Figures 2E,F) were subsequently generated to visualize the resulting gene expression patterns in each dataset.

### 3.3 Functional enrichment analysis of DEGs

**FIGURE 3:**
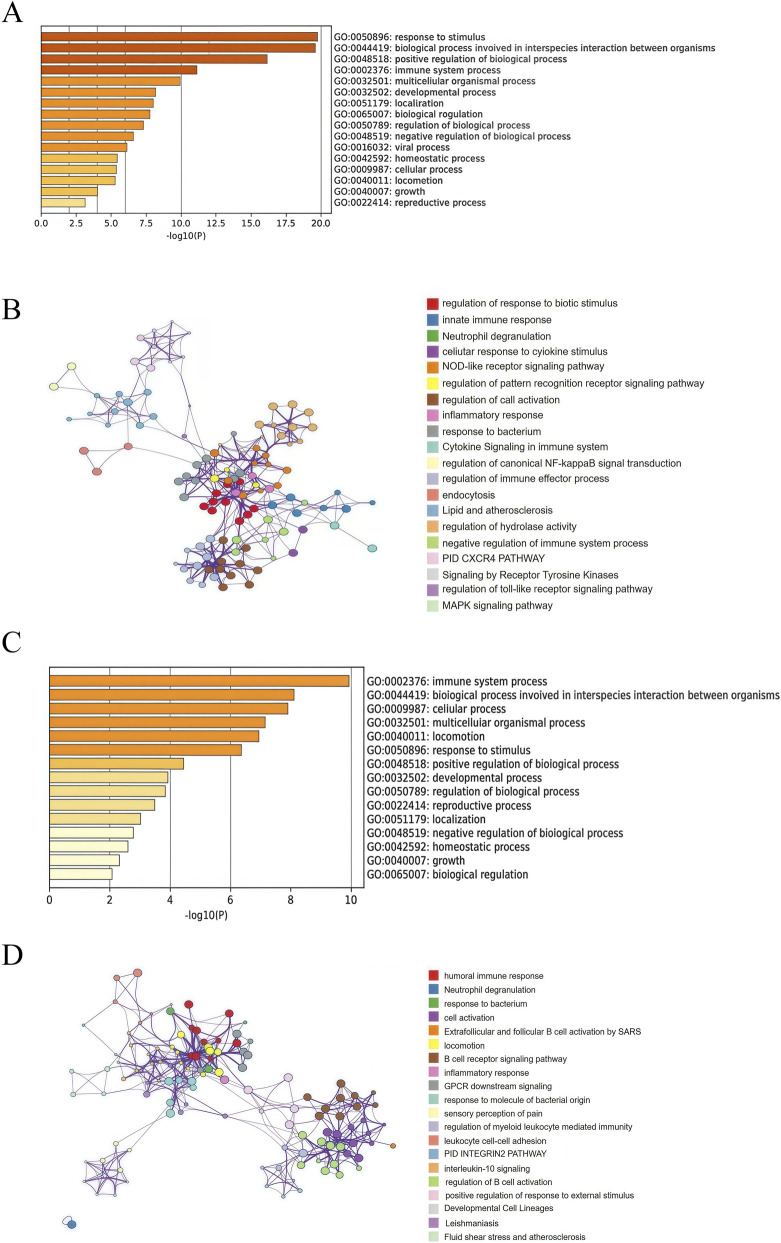
Functional enrichment analysis. **(A)** Bar plot displaying the top 20 significantly enriched GO terms for the PD (GSE10334) dataset. **(B)** Network diagram of enriched GO terms for PD (GSE10334) dataset. Nodes represent distinct GO terms, connecting edges indicate term-term interconnections, while node color and size reflect the enrichment significance. **(C)** Bar plot of GO enrichment analysis for the IE (GSE29161) dataset, highlighting terms associated with cellular processes, immune response, and other relevant categories. **(D)** Network visualization depicting complex relationships among significantly enriched GO terms identified in the IE (GSE29161) dataset.

Functional enrichment analysis was conducted to explore the biological functions associated with DEGs in both PD and IE datasets. Using a significance threshold of *p* < 0.05, significantly enriched GO terms were identified. GO analysis of the PD dataset demonstrated that DEGs were mainly enriched in terms related to response to stimulus, both positive and negative regulation of biological processes, immune system processes, multicellular organismal processes, biological regulation, viral processes, neutrophil degranulation, cellular response to cytokine stimulus, inflammatory response, and response to bacterium, among others (Figures 3A,B).

Similarly, biological process analysis of the IE dataset revealed significant enrichment in immune system processes, multicellular organismal processes, response to stimulus, regulation of biological processes (both positive and negative), homeostatic processes, biological regulation, humoral immune response, neutrophil degranulation, B cell receptor signaling pathway, and interleukin-10 signaling etc (Figures 3C,D).

### 3.4 Determination of the shared hub genes between PD and IE datasets

**FIGURE 4:**
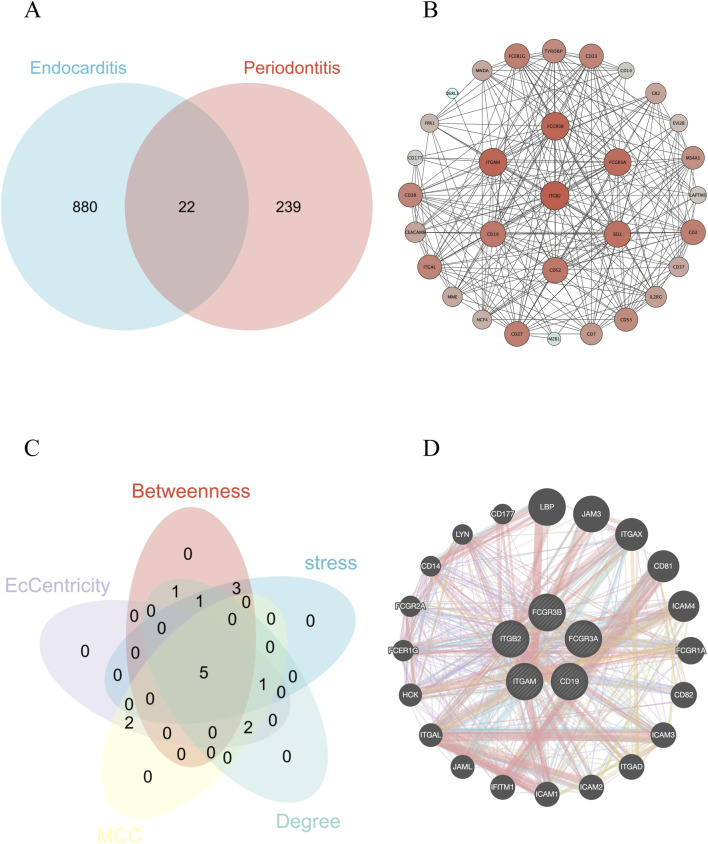
Identification of hub genes. **(A)** Venn diagram illustrating the intersection of DEGs between PD and IE. **(B)** PPI network constructed using the intersecting genes. Nodes represent genes, edges represent interactions between genes. Node size is proportional to centrality within the network. **(C)** Venn diagram demonstrating the screening of core genes using multiple network centrality metrics, ultimately identifying five key regulatory genes. **(D)** Final core gene PPI network highlighting robust interactions among the five key hub genes and their central positions within the regulatory network.

To identify hub genes shared between PD and IE, we intersected 239 DEGs from the PD dataset with 880 DEGs from the IE dataset, yielding 22 common genes (Figure 4A). These shared DEGs were then used to construct a PPI network via the STRING database, which was visualized and analyzed in Cytoscape (Figure 4B). Using the CytoHubba plugin, the top 10 hub genes were identified based on high connectivity scores across five algorithms: Degree, Stress, MCC, EcCentricity, and Betweenness. Other centrality measures, including Diameter, Average Distance, Closeness, Eigenvector Centrality, Bridging Centrality, and Centroid value, were excluded due to the absence of intersecting hub genes. A Venn diagram further refined the candidate set to five high-confidence hub genes: integrin subunit alpha M (ITGAM), Fc gamma receptor IIIb (FCGR3B), Fc gamma receptor IIIa (FCGR3A), integrin subunit beta 2 (ITGB2), and CD19 molecule (CD19) (Figure 4C). Potential interactors of these hub genes, such as lipopolysaccharide binding protein (LBP), junctional adhesion molecule 3 (JAM3), integrin subunit alpha X (ITGAX), CD81 molecule (CD81), and Fc gamma receptor Ia (FCGR1A), were also identified within the PPI network (Figure 4D).

### 3.5 The expression validation of hub genes in the PD and IE datasets

**FIGURE 5:**
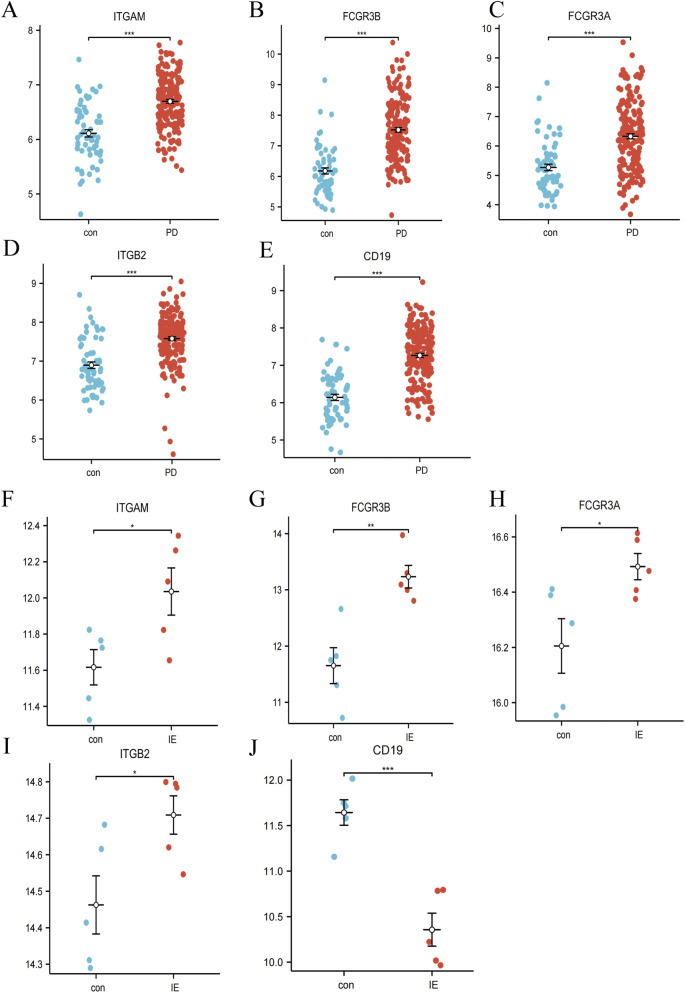
Expression levels of key genes in PD and IE datasets. **(A–E)** Boxplots displaying differential expression of ITGAM, FCGR3B, FCGRA, ITGB2 and CD19 between healthy controls (Con) and PD patients. **(F–J)** Boxplots showing differential expression of ITGAM, FCGR3B, FCGRA, ITGB2, and CD19 between healthy controls (Con) and IE patients. *P < 0.05; **P < 0.01; ***P < 0.001.

To validate the expression patterns of the selected hub genes, we assessed their transcript levels in PD and IE cohorts. In PD patients, all five hub genes (ITGAM, FCGR3B, FCGR3A, ITGB2, and CD19) exhibited significant upregulation compared to healthy controls (Figures 5A–E). Similarly, in the IE cohort, elevated expression was observed for ITGAM, FCGR3B, FCGR3A, and ITGB2, whereas CD19 was notably downregulated relative to normal individuals (Figures 5F–J). Therefore, we selected ITGAM, FCGR3B, FCGR3A, and ITGB2 as potential diagnostic biomarkers for both diseases.

### 3.6 The ROC curve analysis of hub genes in the PD and IE datasets

**FIGURE 6:**
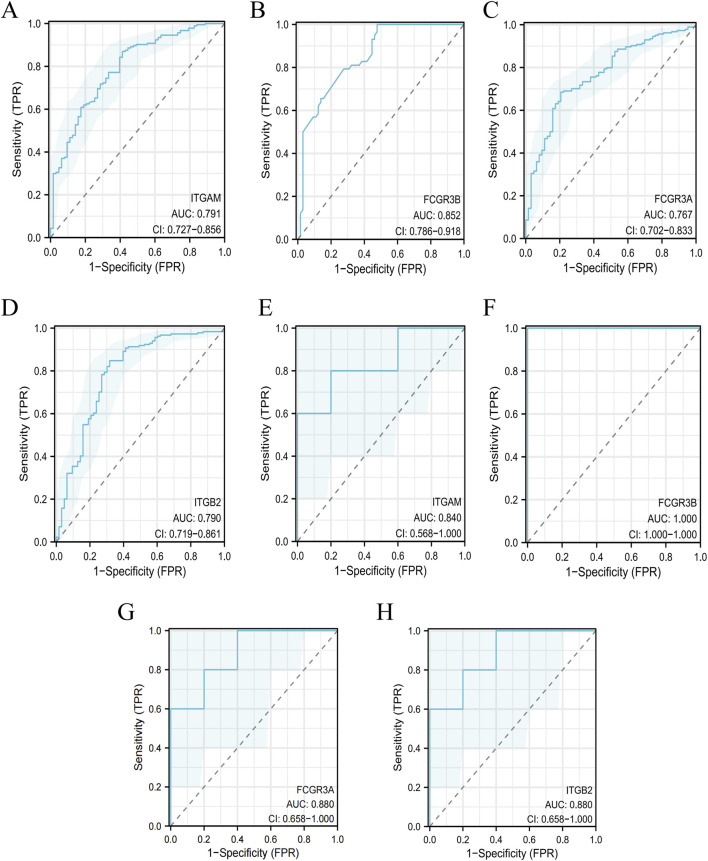
Diagnostic potential of key genes in PD and IE datasets. **(A–D)** Receiver Operating Characteristic (ROC) curves evaluating the diagnostic performance of ITGAM, FCGR3B, FCGRA and ITGB2 in PD. **(E–H)** ROC curves demonstrating the diagnostic performance of ITGAM, FCGR3B, FCGRA, and ITGB2 in IE.

Subsequently, we constructed a diagnostic prediction model based on the GSE10334 (PD) and GSE29161 (IE) datasets. ROC curves were generated to evaluate the specificity, sensitivity, and diagnostic potential of the five hub genes in PD and IE. The results demonstrated promising diagnostic performance for these genes in both disorders. Specifically, in the PD dataset, the AUC values for ITGAM, FCGR3B, FCGR3A and ITGB2 were 0.791, 0.852, 0.767 and 0.790, respectively (Figures 6A–D). In the IE dataset, the corresponding AUCs were 0.840, 1.000, 0.880 and 0.880 (Figures 6E–H). These findings suggested that the four hub genes may serve as potential biomarkers for the accurate diagnosis of PD and IE.

### 3.7 Immune infiltration analysis in the PD and IE datasets

**FIGURE 7:**
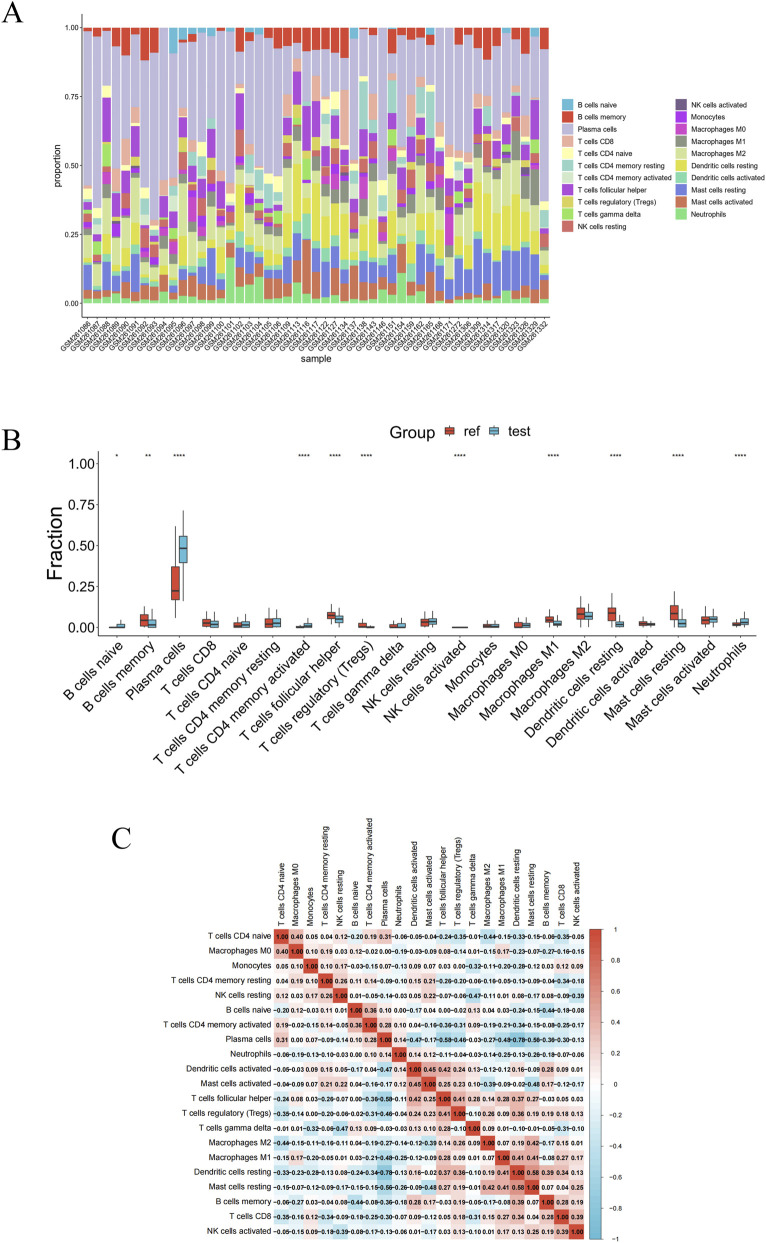
Immune cell infiltration analysis of PD (GSE10334) dataset. **(A)** Stacked bar plot depicting the immune cell subtype composition per sample in the PD (GSE10334) dataset. **(B)** Boxplots demonstrating significant abundance differences of immune cell subtypes between reference (Ref) and test (Test) groups in the PD (GSE10334) dataset. **(C)** Heatmap showed the correlations among immune cells in the PD (GSE10334) dataset. *P < 0.05; **P < 0.01; ****P < 0.0001.

**FIGURE 8:**
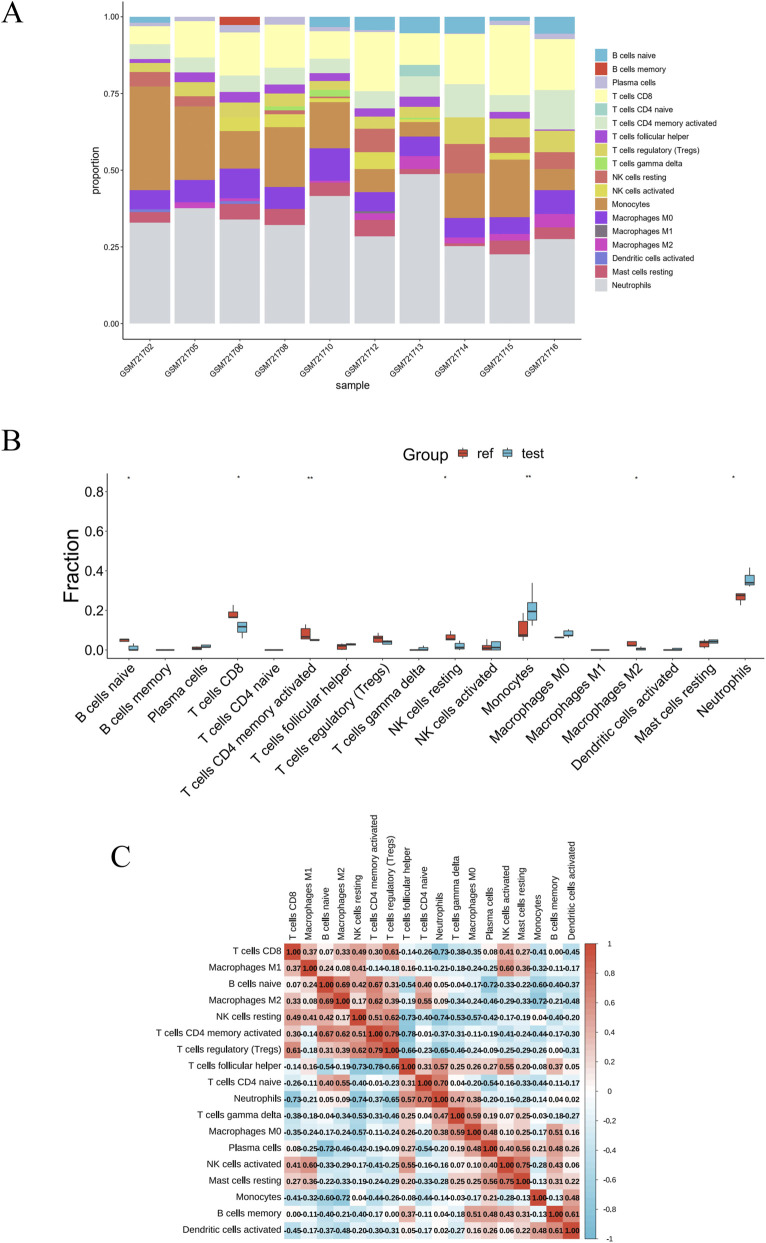
Immune cell infiltration analysis of IE (GSE29161) dataset. **(A)** Stacked bar plot illustrating the compositional proportions of distinct immune cell subtypes across individual samples in the IE (GSE29161) dataset. **(B)** Boxplots comparing the abundance of each immune cell subtype between reference (Ref) and test (Test) groups in the IE (GSE29161) dataset. **(C)** Heatmap showed the correlations among immune cells in the IE (GSE29161) dataset. *P < 0.05; **P < 0.01.

PD is a complex immune-related disorder in which immune infiltration plays a critical role in disease initiation and progression (Kaymaz and Beikler, 2019; Xu et al., 2023). Likewise, immune cell infiltration has been closely associated with IE (Mathé et al., 2024; Brown and GE, 1998). To investigate immune infiltration patterns in both conditions, we performed Immune infiltration analysis using the GSE10334 (PD) and GSE29161 (IE) datasets to quantify the abundance and composition of infiltrating immune cells. In the PD dataset, we observed a significant decrease in memory B cells, follicular helper T cells, regulatory T cells, M1 macrophages, resting dendritic cells, and resting mast cells, along with a marked increase in naïve B cells, plasma cells, activated CD4^+^ memory T cells, and neutrophils in PD patients compared to controls (Figures 7A,B). Similarly, in the IE dataset, reductions were detected in CD8^+^ T cells, naïve B cells, resting NK cells, activated CD4^+^ memory T cells, and M2 macrophages, while monocytes and neutrophils were elevated (Figures 8A,B). Notably, both datasets revealed a consistent upregulation of neutrophils, suggesting a potential common mechanism of neutrophil-mediated immune infiltration in PD and IE.

Subsequent analysis of the immune cell correlation heatmap revealed a positive correlation between resting dendritic cells and resting mast cells (r = 0.58), and a negative correlation with plasma cells (r = −0.78), respectively, in PD samples (Figure 7C). In IE samples, a strong positive correlation was observed between regulatory T cells and activated CD4^+^ memory T cells (r = 0.79). Conversely, a significant negative correlation was identified between follicular helper T cells and the same activated CD4^+^ memory T cell population (r = −0.78) (Figure 8C). These findings pointed to a clear divergence in immune profiles between patients and controls, along with evident crosstalk among various immune cell types.

### 3.8 Correlation between the hub genes and infiltrating immune cells

**FIGURE 9:**
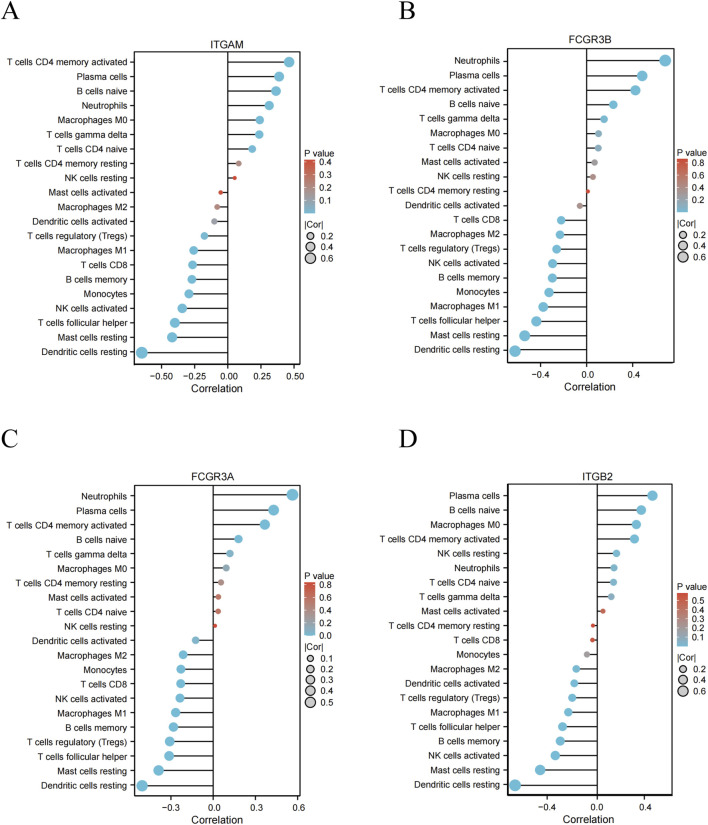
Correlation analysis between key genes and immune cell types in the PD (GSE10334) dataset. **(A–D)** Each panel illustrated the correlation between a specific immune cell subtype and gene expression. Lollipop plots displayed Spearman’s correlation coefficients for immune cell type-gene relationships in the PD (GSE10334) dataset, sorted by the absolute value of correlation strength. Positive and negative correlations were color-coded (blue: negative correlation, red: positive correlation).

**FIGURE 10:**
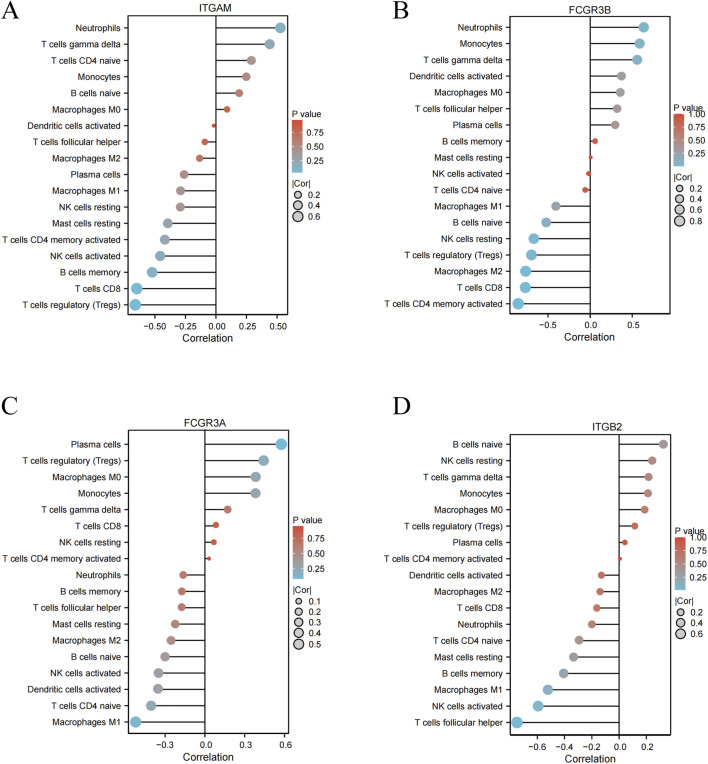
Correlation analysis between key genes and immune cell types in the IE (GSE29161) dataset. **(A–D)** Each panel illustrated the correlation between a specific immune cell subtype and gene expression. Lollipop plots displayed Spearman’s correlation coefficients for immune cell type-gene relationships in the IE (GSE29161) dataset.

The compositional similarity of infiltrating immune cells constitutes only one aspect of the common pathogenic mechanisms underlying PD and IE. To further elucidate the immune-related functions of the 4 hub genes, we performed Spearman’s rank correlation analysis using the GSE10334 (PD) and GSE29161 (IE) datasets to assess their associations with immune infiltration levels. The results demonstrated significant correlations between each hub gene and specific immune cell subsets in both PD (Figures 9A–D) and IE (Figures 10A–D), supporting their potential roles in modulating immune responses across both diseases.

### 3.9 Validation of the hub genes in mice with PD

**FIGURE 11:**
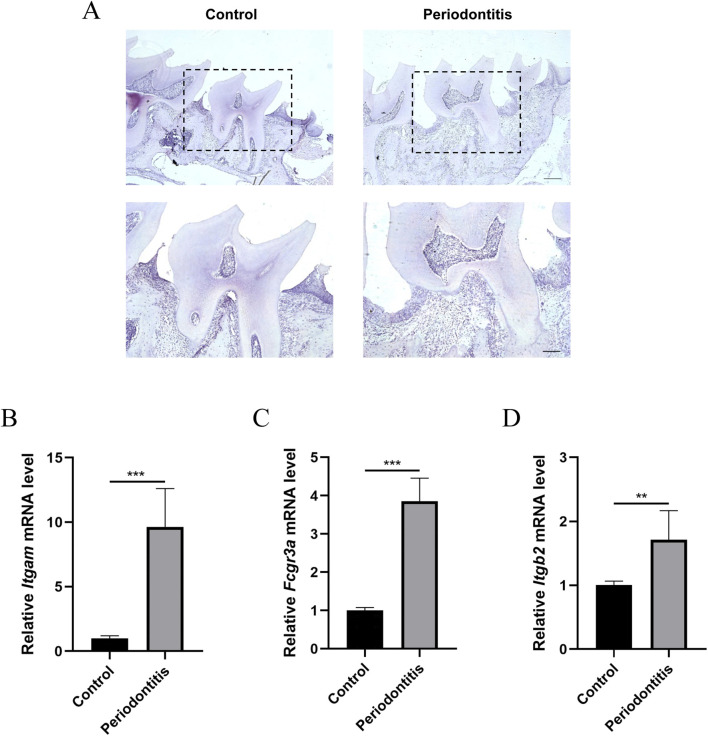
Construction of the PD model and validation of hub genes using RT-PCR. **(A)** HE staining of maxillae in the PD group and the control group (Scale bar, above: 500 μm, below: 250 µm). **(B–D)** The mRNA levels of *Itgam* **(B)**, *Fcgr3*a **(C)**, and *Itgb2* **(D)** in maxillary bone tissues of PD mice compared to the control. **P < 0.01; ***P < 0.001.

**FIGURE 12:**
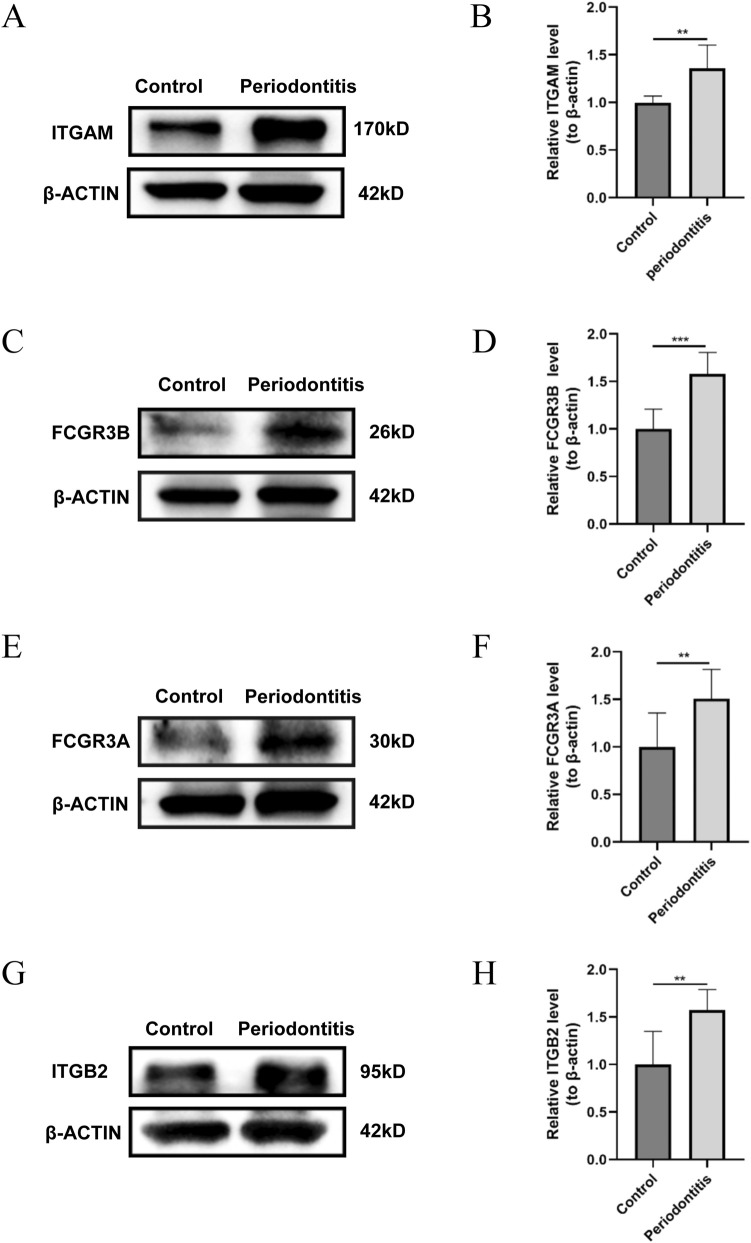
Validation of hub genes expression through WB. **(A–H)** Relative protein expression levels of ITGAM **(A,B)**, FCGR3B **(C,D)**, FCGR3A **(E,F)**, ITGB2 **(G,H)** in maxillary bone tissues of PD and control mice (n = 3 per group). **P < 0.01, ***P < 0.001.

Subsequently, experiments were conducted to validate the expression of the four hub genes. A PD model was established by securing 5–0 silk ligatures around the bilateral maxillary second molars for 6 weeks. The successful induction of PD was confirmed by hematoxylin and eosin staining (Figure 11A). RT-PCR (Figures 11B–D) and Western blotting (Figures 12A–H) analyses revealed significantly elevated expression of the four hub genes in maxillae tissues from ligatured mice compared to the controls.

## 4 Discussion

PD, as a chronic inflammatory disease, is associated with various systemic conditions including cardiovascular diseases (Mei et al., 2020), posing a threat to the health of a growing number of people globally. IE is a cardiovascular disease with a high mortality rate (Lamas, 2020), and PD may have an adverse effect on this condition (Naderi and Merchant, 2020). Although a growing body of evidence indicates an association between PD and IE, the precise mechanisms underlying their co-occurrence have not been fully elucidated. In recent years, bioinformatics has provided novel approaches for analyzing and revealing the pathophysiological mechanism of diseases. Consequently, this study employed bioinformatics to identify shared diagnostic biomarkers for PD and IE, thus offering new insights into the prevention and treatment of these two diseases.

In this study, we initially conducted differential expression analysis on the GSE10334 and GSE29161 datasets to identify DEGs associated with PD and IE. Subsequently, functional enrichment analysis was conducted separately on the DEGs specific to each disease. To uncover hub genes common to both PD and IE, we applied five algorithms from the CytoHubba plugin in Cytoscape, which led to the identification of ITGAM, FCGR3B, FCGR3A, ITGB2, and CD19 as candidate hub genes. These candidates were further validated through expression level assessment and ROC curve analysis. However, due to inconsistent expression patterns of CD19 across both diseases, we ultimately selected ITGAM, FCGR3B, FCGR3A, and ITGB2 as potential diagnostic biomarkers for PD and IE. Additionally, immune infiltration analysis was performed on both disease datasets using the CIBERSORT algorithm. We further examined the immune cell infiltration patterns in PD and IE sample cohorts and investigated the correlations between the identified potential diagnostic biomarkers and the levels of immune cell infiltration. Notably, neutrophils were consistently upregulated in the sample sets of both diseases.

The integrin subunit alpha M (ITGAM), a gene that encodes the integrin alpha M chain (also known as CD11b), assembles into a complex with the integrin subunit beta 2 chain (Lou et al., 2024). Integrins present in periodontal tissues play key roles in facilitating cell adhesion, preserving the structural stability of tissues, and participating in cellular signaling, modulation of gene expression, and the activation of cytokines (Larjava et al., 2014). Furthermore, numerous studies have shown that ITGAM serves as a hub gene linking PD with various other diseases such as diabetes mellitus type 2 (Luo et al., 2025). Zeng et al. identified ITGAM as not only a key gene linking carotid atherosclerosis and PD, but also as a primary mechanism underlying their interplay through its role in immune and inflammatory responses (Zeng et al., 2022). It was observed that ITGAM modulation led to alterations in the secretion of key inflammatory cytokines such as IL-1β, IL-6, and TNF-α (Hou et al., 2024). Thus, ITGAM may contribute to PD through its involvement in the immune-inflammatory process. Existing studies have indicated that ITGAM modulates the cardiac immune microenvironment in models of sepsis-induced cardiomyopathy, either suppressing or enhancing inflammatory responses depending on the disease stage (Huang et al., 2024). Based on this, it is hypothesized that ITGAM may play a similar immunomodulatory role in cardiac tissue damage caused by IE. Nevertheless, the association of ITGAM with both PD and PTB has not been fully elucidated. Our study demonstrated that ITGAM is consistently upregulated in both PD and IE. This finding was further corroborated in a mouse model of PD, indicating that ITGAM serves as a potential link between PD and IE and represents a theoretical immunotherapeutic target for patients suffering from both conditions.

FCGR3A and FCGR3B, located on chromosome 1q23, encode proteins (FcγRIIIA/CD16a and FcγRIIIB/CD16b, respectively) that mediate the recognition and clearance of pathogens by immune cells such as phagocytes (neutrophils, monocytes) and natural killer cells (Cretin et al., 2023). Therefore, FCGR3A and FCGR3B may influence PD through immune responses. A meta-analysis showed that certain polymorphisms in FCGR3A may elevate the risk of developing PD among individuals of Caucasian descent and the FCGR3B NA1/NA2 polymorphism was associated with aggressive PD (Da Silva et al., 2017). An analysis of Hong Kong Chinese individuals revealed that the FCGR3A rs445509 polymorphism was observed to be negatively associated with severe chronic PD (Da Silva et al., 2017). Evidence suggests that FCGR3A mediates the interaction between humoral and cellular immunity in the defense against periodontal microbiota (Chai et al., 2010; Pavkovic et al., 2018). FCGR3B encodes a low-affinity receptor for the IgG Fc domain and exhibits nearly exclusive expression on neutrophils (Lee et al., 2015). This receptor is critically involved in the activation of neutrophil extracellular traps (NETs) (Fonseca et al., 2018). NETs are increasingly recognized as a pathogenic mechanism in IE (He et al., 2025). Given that FCGR3A and FCGR3B both belong to the FcγRIII family, it is plausible that FCGR3A may indirectly influence the progression of IE and inflammation by cooperating with FCGR3B in the induction of NET. In summary, FCGR3A and FCGR3B may play a potential connecting role in the shared pathogenesis of PD and IE.

ITGB2, also known as the integrin beta-2 subunit or CD18, is the key gene that encodes the CD-18β2 integrin protein (Roos et al., 2023). Abe et al. observed a significant upregulation of ITGB2 in the gingival tissues of PD patients. This finding implicated in the enhanced transendothelial migration of leukocytes and the disruption of intercellular communication, which are key features of the disease (Abe et al., 2011). Later studies showed that mutations in the ITGB2 gene can lead to reduced expression of CD-18β2 integrin. Because of this, key white blood cell functions are disrupted, which impairs the clearance of periodontal pathogens and can lead to severe PD (Tewari et al., 2017). The upregulation of ITGB2 in PD was established in our disease model and confirmed by RT-PCR and Western blot analyses. As inflammatory cells, white blood cells may participate in the inflammatory response following endocardial infection by directionally accumulating at the sites of infection and mediating the anti-infection process. Therefore, they play a crucial part in the interplay between inflammation and infection during the development and progression of IE (Thuny et al., 2014). These findings imply a common pathogenic role for ITGB2 in both PD and IE, potentially mediated through similar pathological processes. Consequently, studying the role of ITGB2 in inflammatory pathways could offer key insights for designing clinical strategies against these diseases.

To identify immune cells potentially involved in the shared pathogenesis of PD and IE, we conducted an immune infiltration analysis. The results revealed that neutrophils were consistently upregulated in both PD and IE disease datasets. In response to periodontal pathogens, neutrophils migrate to periodontium. While playing a crucial role in host defense via phagocytosis and antimicrobial release, their excessive or prolonged activation can damage gingival and alveolar bone tissues through the release of inflammatory mediators and enzymes, thus driving PD development. Furthermore, PD may lead to the involvement of neutrophils in cardiovascular pathology through systemic inflammatory mediators, including bacteremia and cytokines (Bassani et al., 2023). Research over the last 10 years has also revealed the significant roles of neutrophils in cardiovascular inflammation and repair (Silvestre-Roig et al., 2020). In IE patients, the neutrophil-to-lymphocyte ratio (NLR) is a potential predictor of embolic events, which are serious complications of the disease (Hu et al., 2021). Studies have revealed that neutrophils contribute to IE in multiple ways: not only do they release substances that, while combating bacteria, can induce autoimmune responses, but they also secrete inflammatory mediators that amplify inflammation and promote thrombosis/vegetation. Moreover, their interaction with platelets and fibrin aids bacterial adhesion and vegetation formation, resulting in valvular destruction and flow obstruction (Ai et al., 2022; Van Gool et al., 2022). Furthermore, a recent study revealed that long-standing PD exacerbates myocardial infarction (MI) outcomes through the expansion of scar-related SiglecF^+^ neutrophils within the heart. MI itself triggers acute cardiac inflammation dominated by massive neutrophil infiltration (Wang et al., 2025). These SiglecF^+^ neutrophils can be primed by PD in the bone marrow and fully activated by cardiac-derived factors (Calcagno et al., 2021; Ryu et al., 2022). Alternatively, they may partially originate from hematopoietic stem and progenitor cells (HSPCs) residing in the gingiva, which enter the circulation via reverse transendothelial migration (rTEM) and subsequently colonize the heart (Wang et al., 2025). Consequently, myocardial repair is, to some extent, contingent upon neutrophil function (Curaj et al., 2020). Therefore, SiglecF^+^ neutrophils could be an appealing target against PD and IE. Future studies should focus on elucidating how neutrophils specifically contribute to the pathogenesis of both PD and IE, and how this knowledge can inform the creation of new treatment approaches. A recently published study in Circulation revealed that in PD patients, oral pathobionts and pro-inflammatory B2 cells markedly aggravate myocardial infarction, supporting the “oral-cardiac axis” as a novel disease pathway. Consequently, interventions aimed at PD and its associated oral pathogens could enhance therapeutic outcomes for certain cardiac conditions (Chen et al., 2025).

However, this study has several limitations. Firstly, the sample size of the datasets we used was limited. This study included a small IE sample set because of the challenges in accessing cardiac valve tissues resulting in a lack of large datasets. Future analyses will employ larger sample sizes to make our findings more compelling. Secondly, our analysis lacked clinical data from patients with comorbid PD and IE. Future studies that can include such patient cohorts would be better suited for further research. Finally, the bioinformatics methods we used were not fully advanced. Despite these limitations, we successfully identified four potential biomarkers for PD and IE. Our experiments confirmed their role in regulating the immune response. This provides new strategies for the future prevention and clinical treatment of PD and IE.

## 5 Conclusion

In this study, we identified four diagnostic biomarkers (ITGAM, FCGR3B, FCGR3A and ITGB2) for both PD and IE via bioinformatic analyses and validated their robust diagnostic performance and immune associations. Enrichment and immune infiltration analyses revealed that the shared pathogenesis of these diseases involves immune-inflammatory responses, thereby laying the groundwork for future exploration of treatments targeting patients affected by both conditions.

## Data Availability

The original contributions presented in the study are included in the article/supplementary material, further inquiries can be directed to the corresponding authors.
